# W-Curve Alignments for HIV-1 Genomic Comparisons

**DOI:** 10.1371/journal.pone.0010829

**Published:** 2010-06-01

**Authors:** Douglas J. Cork, Steven Lembark, Sodsai Tovanabutra, Merlin L. Robb, Jerome H. Kim

**Affiliations:** 1 United States Military HIV Research Program, Rockville, Maryland, United States of America; 2 Henry M. Jackson Foundation for the Advancement of Military Medicine, Rockville, Maryland, United States of America; 3 Illinois Institute of Technology, Chicago, Illinois, United States of America; 4 Workhorse Computing, Woodhaven, New York, United States of America; 5 Division of Retrovirology, Walter Reed Army Institute of Research, Rockville, Maryland, United States of America; Virginia Tech, United States of America

## Abstract

**Background:**

The W-curve was originally developed as a graphical visualization technique for viewing DNA and RNA sequences. Its ability to render features of DNA also makes it suitable for computational studies. Its main advantage in this area is utilizing a single-pass algorithm for comparing the sequences. Avoiding recursion during sequence alignments offers advantages for speed and in-process resources. The graphical technique also allows for multiple models of comparison to be used depending on the nucleotide patterns embedded in similar whole genomic sequences. The W-curve approach allows us to compare large numbers of samples quickly.

**Method:**

We are currently tuning the algorithm to accommodate quirks specific to HIV-1 genomic sequences so that it can be used to aid in diagnostic and vaccine efforts. Tracking the molecular evolution of the virus has been greatly hampered by gap associated problems predominantly embedded within the envelope gene of the virus. Gaps and hypermutation of the virus slow conventional string based alignments of the whole genome. This paper describes the W-curve algorithm itself, and how we have adapted it for comparison of similar HIV-1 genomes. A treebuilding method is developed with the

W-curve that utilizes a novel Cylindrical Coordinate distance method and gap analysis method. HIV-1 C2-V5 env sequence regions from a Mother/Infant cohort study are used in the comparison.

**Findings:**

The output distance matrix and neighbor results produced by the W-curve are functionally equivalent to those from Clustal for C2-V5 sequences in the mother/infant pairs infected with CRF01_AE.

**Conclusions:**

Significant potential exists for utilizing this method in place of conventional string based alignment of HIV-1 genomes, such as Clustal X. With W-curve heuristic alignment, it may be possible to obtain clinically useful results in a short time—short enough to affect clinical choices for acute treatment. A description of the W-curve generation process, including a comparison technique of aligning extremes of the curves to effectively phase-shift them past the HIV-1 gap problem, is presented. Besides yielding similar neighbor-joining phenogram topologies, most Mother and Infant C2-V5 sequences in the cohort pairs geometrically map closest to each other, indicating that W-curve heuristics overcame any gap problem.

## Introduction

In this paper we describe our analysis of variable gp120 sequences from HIV-1 using the W-curve. Our long term goal is to find a fast, clinically useful tool for correlating HIV-1 sequences with neutralization data. However, in order to achieve this goal, we first show in this paper that a W-curve phenogram is comparable to a phenogram of gp120 sequences generated by a conventional string-based neighbor joining tool.

### Representing DNA: Chaos and the W-curve

The W-curve was developed in 1993 as a visualization tool for long genomic sequences [1]. It evolved from the Genomic Chaos Game (CGR), which converts a sequence of DNA bases into a 2-D numerical map [2], [3], [4]. The CGR and W-curve are computationally efficient and are effective for visual comparison of sequences [3, 5, 6, 7, 8, and 9].

**Figure 1:**
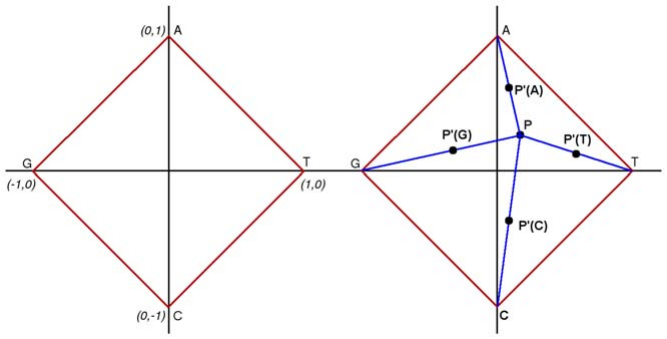
W-curve nucleotide coordinate positions (left) and W-curve projection of each nucleotide (right). The W-curve is generated using a square centered at the origin with corners on the axes (left). Each moving point moves halfway from a starting point P to P' halfway to the corner for the next base in sequence (right). The numbers iterate within the square as follows: P'(T) = [(Px+1)/2, (Py)/2]; P'(A) = [(Px)/2, (Py+1)/2]; P'(G) = [(Px–1)/2], (Py)/2]; P'(C) = [(Px)/2, (Py–1)/2]

The original CGR was generated from the DNA alphabet of {A, C, G, T} by assigning each character to the corner of a square. For example, the original CGR visualization program used the points A→ (−1, 1), C→ (−1, −1), G→ (1, 1), and T→ (1, −1). A slightly different layout was used for our calculations, placing the square with its corners on the axes and T-A-G-C bases at (1, 0), (0, 1), (−1, 0), (0, −1) [Fig. 1, panel A]. This order of corners helps keep the curve from hugging the origin when studying genomic DNA. For example, purines (A/G) and pyrimidines (T/C) are most noticeable in the 2^nd^ and 3^rd^ quadrants. Starting from the origin, the DNA strand is read from the 5′ to 3′, with each base generating a new point in the X-Y plane one half way from the previous point to the corner associated with that base [Fig. 1, panel B]. CGR compares sequences without gaps and mismatches, the distance between them calculated from the difference between the final base points (3′ end nucleotide)coordinates in a CGR [2], [4]. Points in the CGR converge as sequence similarity increases. Almeida et al. [3] verified that CGR's have Markov chain properties.

The W-curve projects the CGR representation along a discrete Z-axis, using base numbers for co-ordinates. Programs for visualizing DNA using W-curves are available for download [10], [11].

Many publications have used the CGR to visualize and evaluate ungapped genes and relatively similar long genomic sequences [12]–[17]. Aligning and comparing whole genomes with W-curves was initially accomplished with visual graphic comparisons [1], [5], [6], [10], [11]. Various distance methods have been employed to compare and align relatively conserved similar sequences [5], [7], [8]. DNA sequences have been shown to exhibit interesting patterns with W-curves and CGRs [1]–[9], [12], [13]. Previous work has been presented that utilize the properties of the CGR to both align and tree various genomic sequences [12], [14], [15]. In addition, preliminary work has been conducted with W-curves to align and tree similar W-curves of genomic sequences. [5], [6], [8].

**Figure 2:**
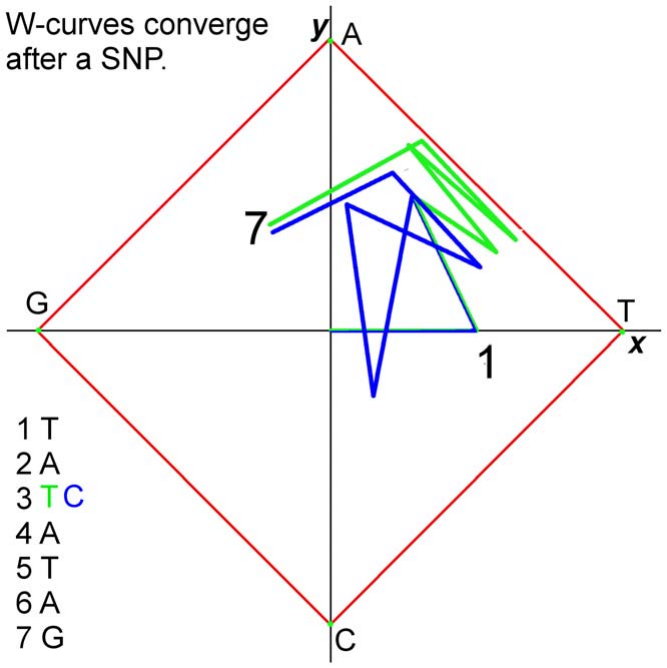
Autoregression characteristics of the W-curve. Two sequences are shown, one in green one in blue. The sequences differ at base 3, which causes the curves to diverge. They converge again by base number 7.

W-curves and CGR's have similar convergence properties. The distinctive difference is that W-curves converge along the Z-axis. Within a few bases of a single nucleotide polymorphism (“SNP”) or at the end of a gap or multi-mer indel, the curves will converge. SNP's leave the curves diverging for short distance, indels a longer one, and gaps cause a phase shift between the curves. Figure 2 is an example of an SNP showing autoregression past a SNP. This is the W-curve for a 7-base sequence with a SNP at base number 3, a T vs. C. The #3 points are in separate quadrants, but by point #5 the curves are close and have similar slopes. A similar effect is seen with gaps in that within a few bases the curves converge.

**Figure 3:**
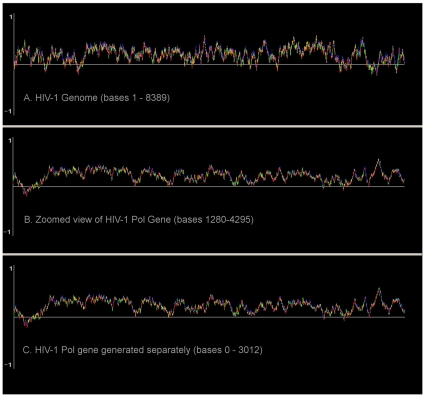
W-curves of a whole HIV-1 genome (A) and embedded pol gene sequence (B, C). A W-curve of an entire genome of HIV-1 01TH.OUR6091 (Accession number AY358040 is shown in panel A. Panel B shows a “zoomed in” projection of pol gene HIV-1 99^TH^.OUR1991 (Accession number AY358039) with respect to base pair position in the whole genome. Panel C shows the same pol sequence extracted from the fasta file and renumbered with respect to base pair position. Sequences can be input into one of two graphical packages for the W-curve existing on the internet (10, 11).

Autoregression also makes it possible to compare curves constructed within a larger sequence against ones generated using a fragment of the sequence. Figure 3 shows an example of the full HIV-1 genome (top), the pol gene portion displayed by zooming in on the bases (middle), and a separate copy of the pol gene generated from a shorter sequence (bottom). After a few bases the two pol gene curves are identical. Convergence between the two pol curves looks identical to a SNP, matching the last base in the genome prior to the pol gene.

**Figure 4:**
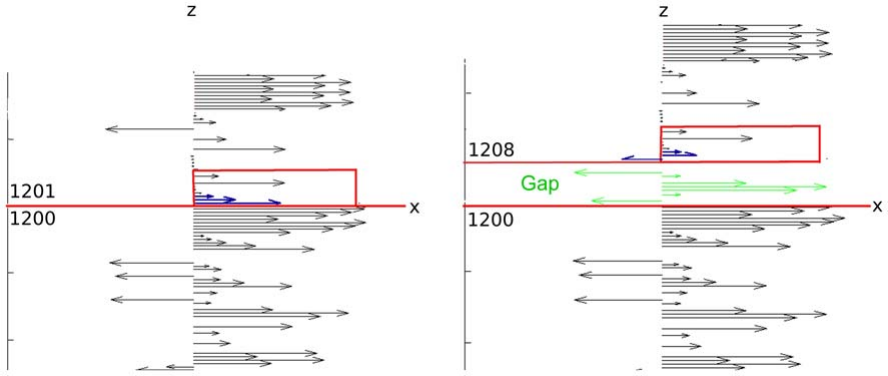
Autoregression in the W-curve after a gap inserted into sequences. The gap (green, right panel) offsets the matching portion of the right curve by seven bases. After three bases for the curves to converge (shown in blue) the curves converge again. Comparing bases 1204 and 1211 (gap + convergence window) will show the curves aligning.

Re-alignment of W-curves after they diverge depends on their autoregressive behavior. Figure 2 shows a regression past a SNP, which only requires examining a few points further down the curve. Larger indels; however, require more work – stepping over an arbitrary piece of curve to find the point of convergence. Gaps further complicate this by introducing a phase-shift between the curves, after which the curves converge [Fig. 4]. Gaps in the W-curve appear as phase shifts between the two curves [Fig. 4]. Reading past the gap in a comparison requires changing the offset of the points being examined. As shown in Figure 4, once the points being compared are re-aligned, the convergence is identical to a SNP or the start of a fragment. The overhead in processing gaps this way is much lower as described in the implementation section. Note that the advantage of the W-curve over the CGR is that local sequential base positions are projected onto the Z axis from the X-Y chaos game plane. This helps to realign similar W-curves with gaps, SNP's and indels, as described in the implementation section below.

Adding the Z-axis gave W-curves the ability to handle sequences with local divergences. This is necessary to handle gaps, which introduce a phase shift (offset in Z-axis value) between the curves after the gap.

The left panel of Figure 4 shows a view of one W-curve in the X-Z plane. The right panel shows the same curve with the addition of a gap at base 1201 (shown in green). After a few bases to re-align the curves (shown in blue) the curves converge in the X-Y plane, but at an offset of seven bases along the Z-axis (i.e., comparing points 1210 from the left to 1217 from the right will give near- zero difference).

**Figure 5:**
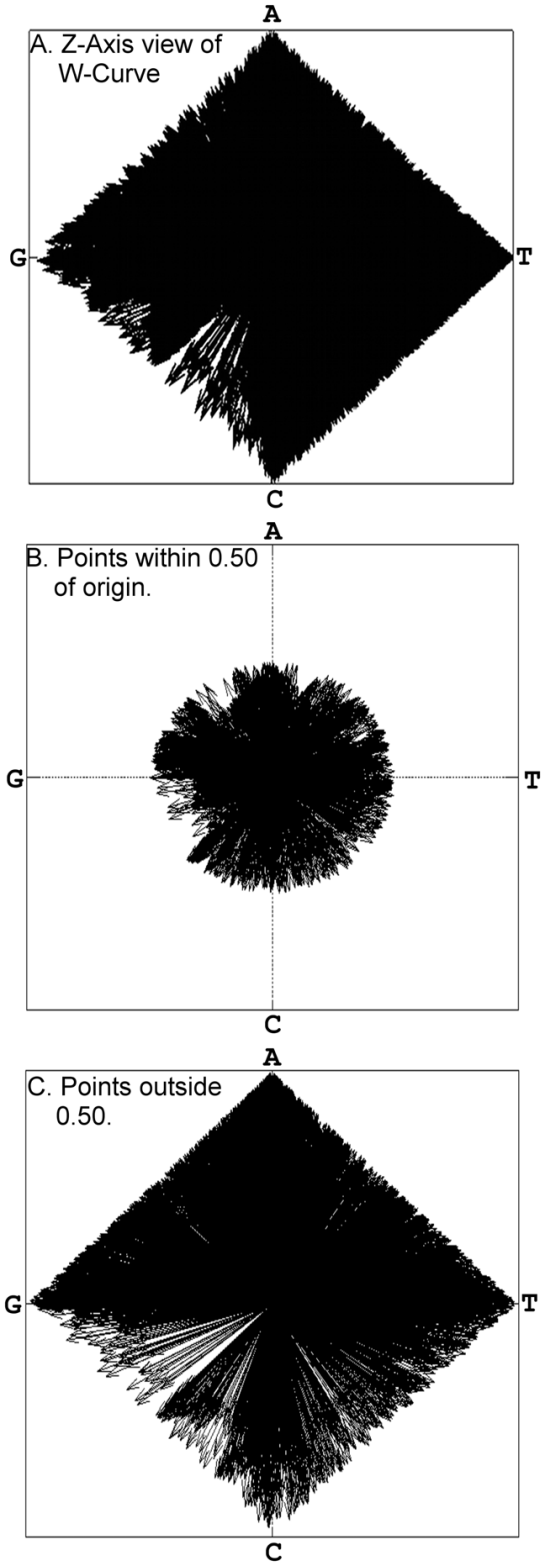
W-curve Z-Axis view of complete HIV-1 Genome showing all points (A), points near origin with radius <.5 (B) and further out, with radius >.5 (C). The last group of points (C) is used to re-align the curves after a gap. CG dinucleotide deficiencies are seen in B and C.

When comparing genomic DNA, the extreme nucleotide points offer a better view of the differences between sequences than ones near the origin. Panels A, B and C of Figure. 5 shows plots of the points in a W-curve plotted radially, viewed along the z-axis. The first panel shows all of the points, and illustrates the well known CG dinucleotide deficiency in the HIV-1 Genome. The second panel shows only points with a radius less than 0.50. The last panel shows only points with a radius >0.50.

These peaks are useful for re-aligning curves after a gap, as they are more discriminating than points closer to the origin and are also sparse. This leaves us with fewer accidental local curve alignments and also fewer points to examine.

### Computations with the W-curve

**Figure 6:**
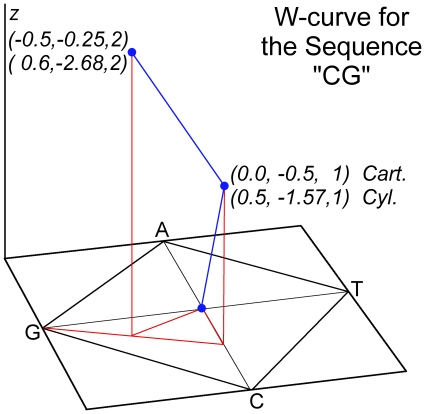
The W-curve for “CG” showing both Cartesian & Cylindrical notation for the points. The curve is shown in blue, layout lines indicating the X-Y locations and line for half-distance rule between the points for C & G are shown in red.

This study used the W-curve for numeric comparison of sequences. The main difference with the visualization programs was rotating the square to have its vertices on the axes [Fig. 1]. With this approach all curves begin at (0, 0, and 0) and have a first point at +/− 0.5 on the X or Y axis, with succeeding points continuing to grow along the Z-axis. We have found that storing the curves using Cylindrical notation simplifies some operations and provides a simple, efficient comparison measure for the curves. Storing the curves this way leaves each point with a radius, angle, and Z-axis value [Fig. 6]. This leaves the first point of each curve with a radius of 0.50 and an angle at some multiple of 90 degrees (π/2).

Comparing the curves requires quickly detecting divergence and smoothing over small differences to locate convergence points. Ideally the difference measure should detect divergence within one base and account for small differences in the curve to allow the detection of convergence within 2–4 bases. For example, handling the SNP in Figure 2 should result in the detection of divergence at base 3 and convergence by base 7.

**Figure 7:**
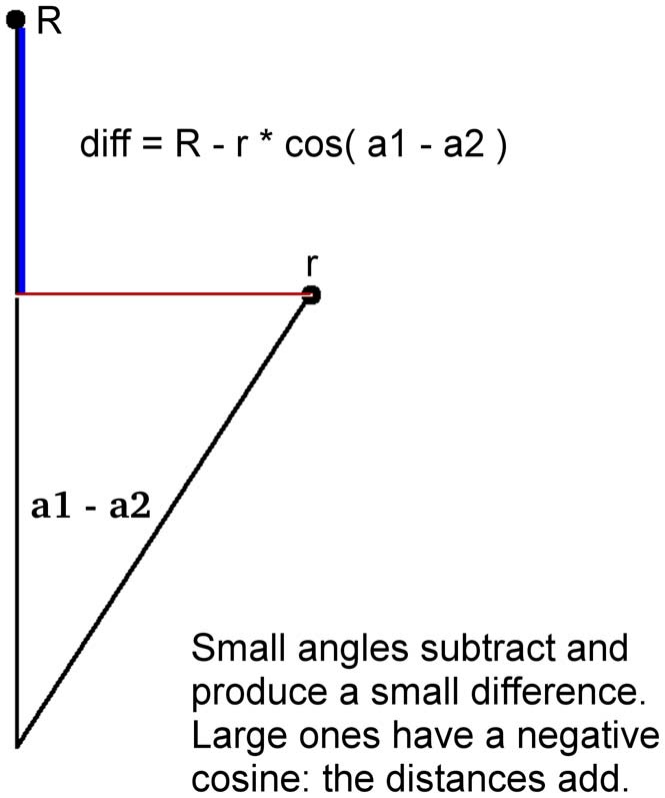
Difference measure for W-curves stored in Cylindrical notation. Subtracting the projection of the smaller radius onto the larger one smooths out small differences after the curves have largely converged. For small angles (shown) the projection is subtracted and produces a small difference. As the angle increases the projection becomes small; points in opposite quadrants have obtuse angles with a negative cosine, adding the projection onto the larger radius.

We have found a simple, fast measure that does both of these. It uses the Cylindrical notation, taking the difference of the larger radius and the smaller one's projection onto it [Fig. 7]. The figure shows the difference for a small angle, where the radii subtract and the difference is relatively small. Once the angle grows past 90 degrees, the cosine goes negative and the distances add. This accentuates divergence into different quadrants, common after a 1–2 base difference while minimizing small differences once the curves have nearly converged

The Cylindrical approach was originally developed for comparing bacteria and was used for an unpublished study of *Clostridia*, which included 8124 species and was the largest neighbor study done to date at 32,995,626 comparisons to generate an upper-triangular comparison matrix. The comparison measure minimizes differences in the same quadrant and accentuates differences in opposite quadrants (e.g., TA vs. CG). In this paper the W-curves are actually stored in cylindrical notation and compared using this measure.

### HIV-1

HIV-1 has a high rate of mutation, in part because it lacks a mismatch repair enzyme. The result is a high rate of gaps and indels that cause problems for most multiple sequence alignment (“MSA”) techniques, including the popular Clustal X [16]. This complicates efforts to correlate the HIV-1 genome with neutralization data, a necessary clinical step in developing effective treatments for HIV-1 infection and AIDS.

As a first step, we have to show that analysis with the W-curve can be used to effectively compare HIV-1 sequences. In our case we compared phenograms generated using the W-curve with others previously done using Clustal X. The process of generating these phenograms is described in the remainder of our paper.

### Data sources

For this publication sequences were taken from a previous report on an HIV-1 cohort study conducted in Thailand [17]. The sequences have GenBank accession numbers EF376993 to EF377287. The sequences themselves are portions of the gp120 including C2 through V5.

## Results

One of our goals was to apply the W-curve to assist researchers correlating viral sequences with clinical outcomes. This will require tuning the process to correctly recognize specific sequences from strains of HIV-1 with known clinical outcomes or correlate regions of the sequences with clinical outcomes as they occur.

In order to accomplish this, we first must show that the clustering of similar W-curves of HIV-1 env genes is congruent with conventional phylogenetic trees generated from these same genes. At the MHRP/HJF, we decided to reexamine C2 thru V5 region of the env gene that was sequenced from a subgroup of eight mother/infant pairs: 051,056,060, 062, 066, 076, 082 and 095. The study was conducted in 1996 and 1997 and C2-V5 sequences were analyzed from HIV-infected infants and their mothers from Lampang Provincial Hospital in Thailand. Cervical/vaginal secretions (CW), ethylenediamine tetraacetic acid plasma (P), and PBMC (Peripheral Blood Mononuclear Cells) were collected from mothers (M) in the third trimester.

**Figure 8:**
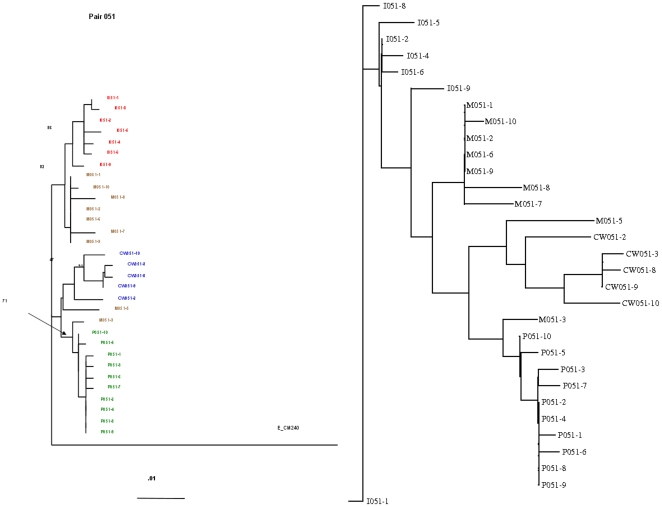
Neighbor-joining string-based phylogenetic tree vs. W-curve-based tree. Envelop C2-V5 from the 051 mother/infant pair grouping from a study previously conducted in Thailand [17]. The string based tree at the left compares the infant sequences to the maternal viruses from different compartments and CM240 as a reference subtype: (I) infant peripheral blood mononuclear cell (PBMC) DNA-derived sequences; M, maternal PBMC DNA-derived sequences; P, maternal plasma-derived sequences; CW, cervical secretion-derived sequences. Numbers at the nodes represent the maximum parsimony bootstrap value. Branch lengths between the sequences are proportionate to the scale bar and indicate the number of mutations per base position per unit time. The tree at the right is the W-curve-based tree. Note the similarity in the clustering patterns. The remaining phenograms are available in the supporting data for this paper [11].

The DNA derived sequences from these 3 different compartments were compared to the infant peripheral blood mononuclear cell (PBMC) DNA-derived sequences (I). CM240 was used as the reference subtype. Phylogenetic analysis was conducted using the Neighbor module of the PHYLIP package (version 3.5c) [18]. A neighbor joining tree of each pair was constructed and evolutionary distances between maternal compartments (CW, P and M) and infant (I) for the M/I pair 051 are examined in Figure 8.

Our analysis began with FASTA files of the sequences, labeled by sample type and GenBank accession number. We generated an upper-triangular comparison matrix using the W-curve with the cylindrical storage format, trigonometric comparison method, and scored the results with a simple sum of the differences and gaps. In this case, the total of differences was added to the gap sizes, divided by two times the longer sequence length. This was done to normalize the difference per gap to 1.0 with the trigonometric approach. The resulting matrix was processed by PHYLIP version 3.67-a neighbor with neighbor-joining and no outgroup or randomization on a single dataset (18). The neighbor output was processed by drawgram with default settings to produce the diagram in Figure 8 (right tree).

The results of both approaches are functionally equivalent. Neighbor, without a molecular clock using the W-curve matrix as input, places the sequences in the same clades. Both techniques also placed the M/I pair M051-3 in between the CW and P samples.

This study also gave us the chance to compare various computational approaches we have developed (see implementation section below). The phenogram in Figure 8 was generated using a W-curve stored in cylindrical notation with a trigonometric comparison. Another analysis of the entire study used Cartesian co-ordinates with a simple sum-of-squares difference. That one correctly assigned the study groups into clades with less time, memory, and code but did not do as well at the detailed distance clustering level, as compared to the string-based trees. In the end, we may find that different graphic approaches are useful at varying levels of detail, offering the flexibility to choose approaches appropriate to the immediate task and resources.

Given our goal of clinical application, reasonable running times on commodity hardware are also important. The largely-single pass nature of W-curve generation and comparison proved to be reasonably fast when run on a desktop system. Table 1 summarizes the comparison size and run times for generating the upper-triangular comparison matrixes we processed with neighbor. These times give a good idea of how well the code performs on commodity hardware. The range of 2–24 sec is well within the clinical requirements for this kind of analysis.

**Table 1:** Benchmark results for sequence comparison using the W-curve.

Group	Sequences	Comparisons	Elapsed	Seq. Rate	Avg. Bases	Base Rate
(A)	(B)	(C)	(D)	(E)	(F)	(G)
		= (B * B+1)/2		= C/D		= C * F/D
051	31	496	2.1 sec	236 Hz	617	4515464 Hz
056	32	528	6.5 sec	80 Hz	596	1528012 Hz
060	40	820	24.3 sec	33 Hz	627	845278 Hz
062	39	780	11.0 sec	71 Hz	611	1696982 Hz
066	36	666	10.4 sec	64 Hz	607	1406507 Hz
076	38	741	2.0 sec	369 Hz	599	8400847 Hz
082	37	703	9.0 sec	78 Hz	607	1754569 Hz
095	40	820	15.2 sec	53 Hz	589	1268291 Hz
Avg	36	694	10.1 sec	123 Hz	606	2676994 Hz

For each group, the number of sequences determines how many comparisons must be made, average number of bases in each sample can be used to estimate the processing rate in terms of the sequence lengths (vs. count of sequences). These times were taken from processing the upper-triangular distance matrices processed by neighbor and drawgram to produce the phenograms from the Mother/Infant study data [11]. Variations in rates are largely due to timesharing overhead, which reflects the likely environment of any clinical application. The data was measured using Perl-5.10.1 on linux-2.6.31 on an Asus M3N-HT (i.e., commodity desktop) motherboard with 4GB RAM and AMD Phenom 3.0 GHz processor.

### Design & Implementation

Our implementation uses Perl for all of the data handling and calculations. The W-curves themselves are stored as linked lists to simplify node-by-node comparison and simplify the addition of skip-chains. We have tried storing the curves in cylindrical and Cartesian notation for use with different scoring algorithms. At this point the cylindrical approach provides better detailed analysis but the Cartesian method is faster and can probably be improved over time. The entire process is broken into two steps: comparison and scoring. The former generates a list of differences between curves in a standard format; the latter generates the values used for display of neighbor joining phenograms.

Like many other languages today, Perl does not support C-style pointers, but it does have references. One issue with comparing W-curves is the need to handle successive pairs of points, possibly offset, one from each curve. Using arrays with integer offset is inefficient for this kind of sequential access, so we used linked lists. The lists also make adding skip-chains trivial, which helps in re-aligning after gaps.

In each case the W-curve starts at the origin: (0, 0, 0). Since most DNA sequencing systems use base counts instead of offsets this keeps the subsequent Z-axis values in sync with the base numbers. Storing the W-curve requires saving the half-interval points, combined with the current base number for a total of three values.

### Skip-Chains

**Figure 9:**
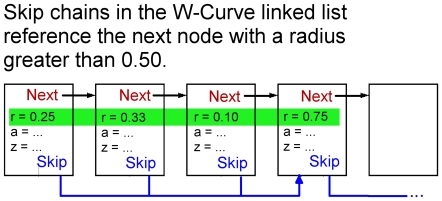
Linked List and Skip Chain. Points on the W-curve are stored as nodes in a linked list, using either Cylindrical (shown) or Cartesian notation. The skip chain for each node references the next node with a radius greater than 0.50, giving direct access to nodes used in re-aligning the curves after a gap or indel.

Our process begins by converting each DNA sequence to a linked list of W-curve points. The list nodes are stored as array references, with a next link followed by the geometry [Fig. 9]. Adding the skip chain starts at the head node, pushing a sentinel node ahead to the next point outside the cutoff distance from the origin. For the example in Figure 9, the fourth node is the first one with a radius greater than 0.50. All the intervening nodes get a reference to the sentinel, which advances the working node up to the sentinel, and the process starts over again. Any links at the tail of the list simply do not get a skip reference added to them.

Comparing the nodes starts with the head of each linked list. At this point the relative offset of the nodes is zero. The initial alignment asks if the curves have a small difference for the first few nodes, if so the initial chunk is inserted and comparison continues; if not then the curves need to be aligned.

With this approach, SNP's show up as a non-zero difference between the curves. If the curves converge after a few bases without adjusting their relative offset then there was an SNP and the comparison can keep going ahead. Due the high rate of mutation in HIV-1, SNP's adjacent to one another – or close enough to keep the curves from converging – are fairly common. As a result, we use a relatively large window for SNP checks: 8 bases. Checking for convergence within that range is less expensive than trying to re-align the curves.

### Re-aligning nodes after a gap

Re-aligning the curves after a gap starts with a Cartesian product of points within a window downstream of the divergence. Taking a window larger than the largest expected gap means that the curves will converge at least once within this gap window. Within the window, pairs of points are examined for convergence for a few bases. If the curves seem to match then the new relative offset is incremented. When all points in the window have been examined the most frequent value is chosen as the new offset and the difference in offsets provides the size of the gap. Examining points past the first match is necessary to avoid issues with extraneous matches. It also gives equal weight to gaps on either sequence.

This is where the skip chain is used: only points further from the origin are used to start comparing the curves for convergence. This saves checking about half the points on average and produces fewer accidental matches due to points simply being close to the origin.

Cylindrical storage leaves the difference a bit less obvious: the angles will be larger but the radii might both decrease. Using the trigonometric approach, a cutoff of 0.25 works well as a test for diverging curves.

Given our goal of flexibility, comparison and scoring are handled separately. The comparison generates values for each contiguous section of W-curve along with one each for the initial alignment and trailing portions of the curves. These comparison values can be stored for later use and provide a standardized interface for the scoring algorithms. In particular, the ability to store comparison results will allow us to quickly evaluate and tune scoring methods for specific cases.

The basic structure used for comparison stores five values for each contiguous section of the W-curve.

The values describe the beginning, end, and SNP difference within the section in four z-axis values and a running total. Due to initial alignment issues, the sections may not start on the same z-axis value. For example, if a section of curve ran from base position 110 → 150 on one curve and 115 → 155 on the other with a total SNP difference of 23.45, the section's data would look like:
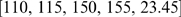



The run lengths of these section are the same, allowing a sanity check of subtracting the starting and ending bases for each curve and checking that they are equal (e.g, 150–110 = 155–115). Another check is that the relative offsets of the bases on each side do not change (e.g., 110-115 = 150–155).

Frequently the curves do not align at the start. This requires starting the comparison from a non-zero z-axis value on one or the other curve – or both in many cases. The initial alignment is stored in the first section. This always begins at 0 on each curve and ends wherever the curves themselves match. For example, if two curves aligned properly at offsets 4 & 5, the initial section would look like:
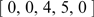



Trailing differences are handled in a similar fashion. If two curves of length 1000 & 1200 were aligned over their entire lengths (i.e., SNP differences only, no gaps) then the final section would look like:
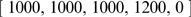



Note that there will always be zeros for the first two positions of the initial section, zero for the SNP difference in the trailing section, and one pair of values on the trailing section will have a run-length of zero. The before-and-after sections are where the run-length sanity check is violated – and also why five values are used to store the section values instead of starting points and run lengths: differences at the beginning and ends of the curves are handled with a constant format.

Looking at the sections of two curves, the simplest case is a curve compared to it. If the sequence is N+1 bases long, the z-axis values as offsets will be (0 ... N). The before section will show no initial offset, the final section will show no trailing difference, and the middle can be handled as a single section with zero total difference between the curves:
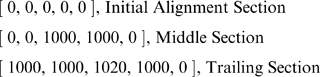



If a pair of the curves 1001 bases long required a five-base offset on one curve to align and have no gaps then their sections would look like:
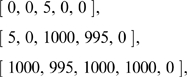



with the final section using up the last five bases on the second curve.

Comparing curves identical aside from 20 trailing bases on one curve would yield sections of:
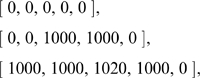



SNP's add to the total difference score within each set. Comparing equally long curves with only SNP differences would give a non-zero SNP total for the main portion:
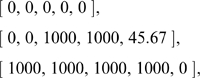



Gaps introduce offsets between the start of one set and another. Two curves align at the start and have a single gap of 20 bases (520–500) in the middle would look like:
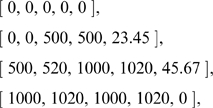



SNP's account for a total difference of 23.45 in the first region, 45.67 in the second one. The last set of numbers shows that the gap accounted for all of the difference in length between the two curves.

Values for curves that did not align at the start, had a few gaps, and were mismatched in size at the end would look like
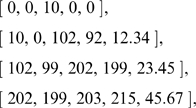



which indicates that the initial alignment started at base position 10 on the first curve, 0 on the second. In 92 bases there was a gap that advanced the second curve (92 → 99). Finally, the second curve is longer since comparison ends at 203 on the first curve, 215 on the second.

We currently normalize the score between curves to an average difference per base in the range of 0 to 1 inclusive. Zero indicates identical curves; one indicates that the curves lived in opposite corners for the entire comparison. For the trigonometric difference calculations, this requires dividing the total difference by two. Gaps are counted with a weight of one in both techniques, with leading alignment and trailing portions of curves treated as gaps. The total of normalized differences and gap scores is divided by the larger curve's length, which is the maximum of the two lengths in the last set of data, in order to get a final difference value.

For the last example, the total length is 215 (i.e., max 203, 215). Differences total 81.46, which would normalize to 40.73 for the trigonometric comparison. Gaps are an initial 10, intermediate 7 (99-92), and trailing 12 (215–203) for a total difference of 40.73+29 = 69.73. Dividing by the larger curve yields a difference of 0.324. There is no question that this comparison is simplistic, but it does work well in practice and splitting up the comparison and scoring will allow us to evaluate multiple scoring rules as we try to improve our algorithms.

### Availability and Future Directions

Initial versions of the W-curve code and data used for the analysis presented here are available as a single download from the W-curve's URL [11]. The code is currently somewhat elementary but should serve as a starting point for anyone interested in our approach. Recent research with the CGR tool has led to a defined spectrum of genomic signatures [19], a CGR of protein sequences [20], a 3D graphical representation of RNA secondary structures[21], and further comparisons of whole genomes [7], [15]. In addition, going forward, our immediate goal is applying the W-curve for clinical use in treating HIV-1. We are working in two main areas here: improving the accuracy of our scoring algorithms and automating analysis of genomic sequences from patient samples. Improved comparison will require simply trying various approaches to see which ones best models the clinical similarity between HIV sequences. Automation will require modifying our comparison and scoring algorithm to account for large gaps in order to compare fragments of curves to genome sequences from patient samples.

The first improvements we can make on comparing sequences will be developing a workable Cartesian approach for comparing the curves. The Cartesian approach is desirable for speed and also because it lends itself to use with integer mathematics, which helps both speed and memory use. This is where the graphical approach has an advantage over other techniques: the flexibility in analysis tools allows us to adjust the outcomes to match the foibles of HIV-1.

The current level of matching is sufficient to allow automating more detailed analysis of HIV-1 genetics and clinical data. Current research on HIV-1 is looking for ways to reliably predict neutralization based on the phylogenetic clustering of the sequences as well as statistical analysis of the evolutionary distances between the entire genome, genes, or even large proteins [22]–[27]. There appears to too much noise to make reliable predictions. More recent studies have focused on specific regions of the env gene. The important area is gp120, which has a mix of variable and conserved regions (C1 thru V5 of the env gene). The question is can they be correlated to immune function. The Maternal/Infant study described in our results looked at gp120's C2-V5, but even this region may include some unnecessary variable regions. The problem is that even the locations of each fragment within gp120 vary between samples, requiring significant manual curation to prepare the sequences for comparison.

Examining a Cartesian product of points works, but is expensive. Peaks help, but are not enough for large windows necessary to handle larger gaps or indels. Two approaches for mitigating the cost would be progressive comparison and some other algorithm like nearest-neighbor to estimate the comparison points. Progressive scanning would use some cutoff based on minimum vote count and ratio to the next-best estimate; other algorithms could be used to reduce the number of points that have to be examined.

### Building a fragment library

**Figure 10:**
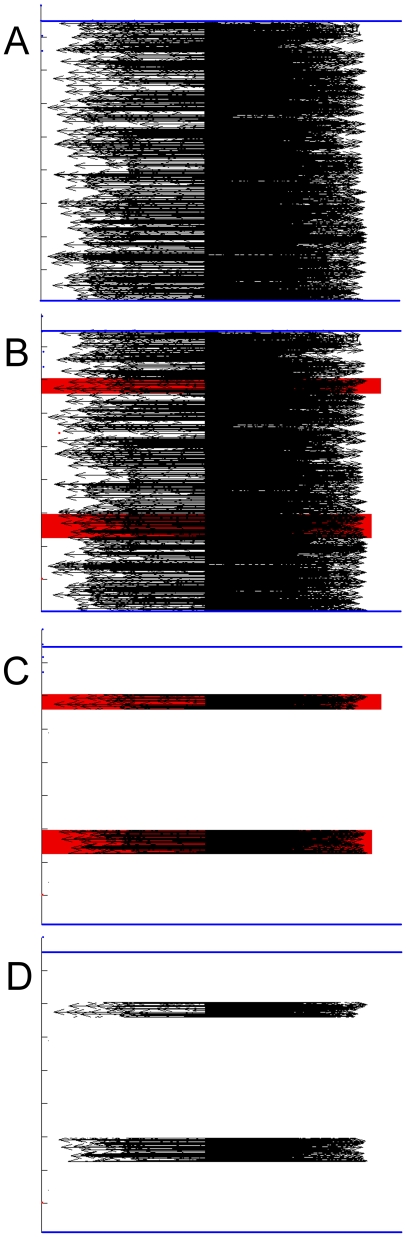
Building a fragment library. Panels A, B, C, D describe the process of extracting curve fragments. Autoregression allows re-use of the curve fragments for comparisons between curves. Starting with the W-curve for a genome or gene (A), regions corresponding to the sequences of interest are found (B) and the remainder of the curve dropped (C), leaving a set of smaller curves (D). The set of curve fragments can be used to search for a list of regions in or score only part of a gene. For example, scoring only the conserved regions of gp120 may prove more effective for generating phenograms than using the entire gp120 sequence or *env* gene.

The W-curve can help here because fragments of curves can be matched easily. As shown in Figure 10 due to autoregression, regions of gp120 in a W-curve from a genome or gene sample can be compared to a library of fragments taken from samples with known clinical outcomes. This allows for quick identification of both the location of these regions on the sample and generating comparison scores for the fragments.

Where the current studies compare contiguous regions of gp120, this approach would generate individual scores for each conserved and variable region on the samples. This approach should remove extraneous variability in the sequences being compared with their clinical outcomes.

This process would start with the W-curve for an entire genome [Fig. 10-A]. We can then identify the regions of interest [Fig. 10-B, in red], drop the remainder of the curve [Fig. 10-C], which leaves us with fragments [Fig. 10-D] that can be matched to other genome or whole-gene curves.

This should enable us to meaningfully compare the curves, even when there will be huge gaps in the comparison. Hopefully this will give us a repeatable process for predicting the neutralization outcome of known vaccines.

The process for this analysis would begin with a set of gp120 sequences for standard genomes whose gp120 sequence have already been broken down into conserved and variable regions in both local (env) and global (whole genome) regions of HIV-1. From there we can generate W-curves for the individual conserved and variable regions. We would then use our existing analysis to compare the regions with the new samples, adjusting the scoring process to account for what otherwise look like huge gaps around the fragments. If we can identify the conserved region locations, we can use that knowledge to correctly locate and compare the variable regions. The technique mimics virtual immunoprecipitation or hybridization with same-sense W-curves rather than anti-sense DNA or antibodies.

The advantage to this approach is automation: manual overhead limits present studies to larger, contiguous sequences. The W-curve does not require gap-stripped, reduced sequences to run efficiently. We can already group samples into major clades quickly, adding an integer approach should help speed things up and make even larger studies possible.

One important breakthrough in comparing W-curves will be the addition of indeterminate bases to the process. This might require “forking” the curves or replacing our point comparisons with an area and storing intermediate bases as a volume described by the base members. Either way, handling this data would allow us to directly process the Fastq output of Second-Generation equipment used to process the HIV-1 sequences, such as the Illumina. This approach could use fragments to store the divergent portions of the curve, allowing comparison between curves for closest-match estimates or maximum-likelihood estimates based on the quality scores available in fastq data.

One similar area for fragments would be determining the order of genes with bacteria or quickly identifying dangerous bacteria from samples. Currently, both of these processes are time-consuming and require significant manual effort. A library of fragments could be used to locate the genes in order or determine if smaller fragments are present in the samples.

Our algorithms also lend themselves well to multithreading. Threaded processing of W-curve fragments could lead to very-high-speed analysis of larger sequences or – ideally – fast, fully automated analysis of clinical samples.

W-curves should be viewed as a separate iterated function system family possessing autoregressive properties in the generation of self-similar local and global patterns [28], [29], [30]. These properties, when properly applied by computational biologists and bioinformaticians, have an enormous capacity to generate visual representations that will allow the user to inspect and compare both local and global features in genomic sequences. It may be possible that the conformational changes in these local and global genomic features, when correlated with clinical outcomes due to infection by these sequences may lead to breakthroughs in vaccine and antiviral development.

## References

[1] Wu D, Roberge J, Cork DJ, Nguyen BG, Grace T (1993).

[2] Jeffrey HJ (1990). Chaos game representation of gene structure.. Nucleic Acids Res.

[3] Almeida JS, Carrico JA, Maretzek A, Noble PA, Fletcher M (2001). Analysis of genomic sequences by chaos game representation.. Bioinformatics.

[4] Huang G, Liao B, Li Y, Yu Y (2009). Similarity studies of DNA sequences based on a new 2D graphical representation.. Biophysical Chem.

[5] Cork DJ, Marland E, Zmuda J, Hutch TB, Valafar F (2002). Achieving Congruency of Phylogenetic Trees Generated by W-curves of Genomic Sequences.

[6] Cork DJ, Lapointe FJ, McMorris FR, Janowitz MF, Mirkin B, Roberts FS (2003). Achieving Consensus of Long Genomic Sequences with the W-curve..

[7] Cork DJ, Wu D, North MJ (2008). Geometric global genomic informatics for RNA viruses, Int.. J. Medical Engineering and Informatics.

[8] Cork DJ, Toguem A, Valafar F (2002). Using Fuzzy Logic to Confirm the Integrity of a Pattern Recognition Algorithm for Long Genomic Sequences: The W-curve..

[9] Basu S, Pan A, Dutta C, Das J (1992). Mathematical characterization of chaos game representation. New algorithms for nucleotide sequence analysis.. J Mol Biol.

[10] http://mypages.iit.edu/~cork/houstep/introduction

[11] http://www.bioinformatics.org/wcurve/

[12] Hill KA, Schisler NJ, Singh SM (1992). Chaos game representation of coding regions of human globin genes and alcohol dehydrogenase genes of phylogenetically divergent species.. J Mol Evol.

[13] Oliver JL, Bernaola-Galvan P, Guerrero G, Roman-Roldan R (1993). Entropic profiles of DNA sequences through chaos-game-derived images.. J Theor Biol.

[14] Deschavanne PJ, Giron A, Vilain J, Fagot G, Fertil B (1999). Genomic signature: characterization and classification of species assessed by chaos game representation of sequences.. Mol Biol Evol.

[15] Joseph J, Sasikumar R (2006). Chaos game representation for comparison of whole genomes.. BMC Bioinformatics.

[16] Thompson JD, Gibson TJ, Plewniak F, Jeanmougin F (1997). The Clustal X windows interface: Flexible strategies for multiple sequence alignment aided by quality analysis tools.. Nucleic Acids Research.

[17] Tovanabutra S, de Souza M, Sittisombut N, Sriplienchan S, Ketsararat V (2007). HIV-1 genetic diversity and compartmentalization in mother/infant pairs infected with CRF-1_AE.. AIDS.

[18] Felsenstein J (1992).

[19] Wang Y, Hill K, Singh S, Kari L (2005). The spectrum of genomic signatures: from dinucleotides to chaos game representation.. Gene.

[20] Yu ZG, Anh V, Lau KS (2005). Chaos game representation of protein sequences based on the detailed HP model and their multifractal and correlation analyses.. Journal of Theoretical Biology.

[21] Feng J, Wang T (2008). A 3D graphical representation of RNA secondary structures based on chaos game representation.. Chemical Physical Letters.

[22] Fenyö EM, Heath A, Dispinseri S, Holmes H, Lusso P (2009). International network for comparison of HIV neutralization assays: The NeutNet report.. PLoS ONE.

[23] Brown BK, Sanders-Buell E, Rosa Borges A, Robb ML, Birx DL (2008). Cross-clade neutralization patterns among HIV-1 strains from the six major clades of the pandemic evaluated and compared in two different models.. Virology.

[24] Scarlatti G, Albert J, Rossi P, Hodara V, Biraghi P (1993). Mother-to-child transmission of human immunodeficiency virus type 1: correlation with neutralizing antibodies against primary isolates.. J Infect Dis.

[25] Polonis VR, Chanbancherd P, Chantakulkij S, Jugsudee A, Loomis-Price LD (2001). HIV type 1 subtype E-infected patients with broadened, dual (B/E) V3 loop serology have increased cross-neutralizing antibodies.. AIDS Res Hum Retroviruses.

[26] Mascola J, Gilbert P, Hahn BH, Haigwood NL, Morris L (2005). Recommendations for the design and use of standard virus panels to assess neutralizing antibody responses elicited by candidate human immunodeficiency virus type 1 vaccines.. J Virol.

[27] Nyambi PN, Nkengasong J, Lewi P, Andries K, Janssens W (1996). Multivariate analysis of human immunodeficiency virus type 1 neutralization data.. J Virol.

[28] Peitgen HO, Jurgens H, Saupe D (1992).

[29] Barnsley MF (1988). Fractals Everywhere..

[30] Mandelbrot BB (2004). Fractal Geometry of Nature..

